# Copland’s Medical Dictionary

**Published:** 1846-05-01

**Authors:** 


*Copland's Medical Dictionary*.—This comprehensive work has reached the eleventh number. We have already expressed our views of its value, embracing as it does, a library of practical medicine in itself. The additions of the able American Editor are copious, and enhance greatly the usefulness of the work for the American practitioner. On the subject of fever, especially, our attention has been drawn to the extensive contributions of Dr. Lee, who has presented an admirable epitome of the present position of this subject as regards facts and doctrines. We are gratified to see that he has been induced, by request, to transfer his notes to the columns of the New-York Journal of Medicine, thereby affording an opportunity for their perusal by many who will neglect to purchase the Dictionary.

